# A Simple and Rapid LC-MS/MS Method for Quantification of Total Daidzein, Genistein, and Equol in Human Urine

**DOI:** 10.1155/2020/2359397

**Published:** 2020-01-20

**Authors:** Shikha Saha, Paul A Kroon

**Affiliations:** Quadram Institute Bioscience, Norwich Research Park, Norwich NR4 7UQ, UK

## Abstract

Isoflavones and isoflavandiols have shown many health benefits, such as reducing cardiovascular disease, cancer, age-related disease, and osteoporosis. However, to investigate the relationships between consumption of isoflavones and their health benefits, it is important to be able to accurately quantify exposure in the large numbers of samples typically produced in association studies (i.e., several thousands). Current methods rely on solid-phase extraction protocols for sample cleanup, resulting in protracted extraction and analysis times. Here, we describe a fast and easy sample preparation method of human urine samples for subsequent quantification of daidzein, genistein (isoflavones), and equol (isoflavandiol) using LC-MS/MS. Sample preparation involves only the addition of dimethylformamide (DMF) and formic acid (FA) after enzymatic hydrolysis of their metabolites by a *β*-glucuronidase and sulfatase mixture. The method was validated by precision, linearity, accuracy, recoveries, limit of detection (LOD), and limit of quantification (LOQ). Linear calibration curves have been shown by daidzein, genistein, and equol. The correlation coefficients values are *r*^2^ > 0.99 for daidzein, genistein, and equol. LOD for daidzein and genistein was 1 ng/ml and equol was 2 ng/ml. Recoveries were >90%, and the relative standard deviation for intraday (<10%) and interday (≤20% over 10 days) was good. This method is suitable for quantification of isoflavones and the microbial metabolite equol in human urine and is particularly useful where large numbers of samples require analysis.

## 1. Introduction

Isoflavones belong to the polyphenols family of plant secondary metabolites [1] and are common components of the human diet. Daidzein and genistein are isoflavones, and equol is the end metabolite of daidzein produced by the metabolic action of a particular intestinal bacteria [2]. There is much supporting evidence that isoflavones have health-promoting effects and alleviate many diseases [3]; its role in the reduction of hormone-related diseases, cancers, menopausal problems, osteoporosis, and cardiovascular diseases has all been reported [4–7].

Previous studies showed that glucuronide and sulfate conjugates of daidzein, genistein, and equol are the main circulating metabolites in humans [8]. To quantify daidzein, genistein, and equol in human bodily fluids, it is common practice to first hydrolyse samples with *β*-glucuronidase and/or sulfatase enzymes. The hydrolysed products are the aglycons, daidzein, genistein, and equol, which are then quantified against authentic standards [9]. The hydrolysis reaction is affected by pH, temperature, hydrolysis time, concentration, the source of the enzyme, and the kind of sample matrix. After hydrolysis, samples are cleaned up using either a solid-phase extraction (SPE) or a liquid-liquid extraction (LLE) method. A variety of chromatography and detection techniques, for example, HPLC-UV, LC-UV/PDA, fluorescence, mass spectrometry (MS) or coularray electrode array detection [10–12], and gas chromatography with mass spectroscopy detection (GC-MS) [13] have been used for quantification of the aglycone forms. Nowadays, mass spectroscopy is a common detection technique because mass spectroscopy is very sensitive and specific [14–20]. However, sample preparation and sample cleanup before analysis by mass spectroscopy can be very complicated and time consuming; indeed previous studies using LC-MS/MS techniques for the quantification of total daidzein, genistein, and equol in human biological fluids required prolonged hydrolysis and cleanup by SPE [14, 16, 21–23] and LLE [24–28].

Grace and colleagues have reported the quantitative analysis of daidzein, genistein, and equol in human biological samples using LC-MS/MS techniques [14]. However, the authors used the robotic liquid handling SPE technique in their sample extraction method. Another study reported that the LC-MS/MS method used the atmospheric pressure photoionization (APPI) source which is not a common or widely applicable ionisation approach in mass spectroscopy [29]. Also, longer sample preparation times (incubation time of 12 h at 45°C) were required for complete enzymatic hydrolysis of metabolites, which extended the total analysis time and increased the risk of sample instability.

Human cohort studies normally generate a huge number of samples, and fast and sensitive analytical methods are needed. Therefore, our aim was to develop a new method to analyse substantial numbers of human urine samples without requiring (1) sample preconcentration by a SPE/LLE extraction method, (2) high sample volume, and (3) long incubation time for complete enzymatic hydrolysis. Here, we report a method for the analysis of total daidzein, genistein, and equol which allows rapid and accurate quantitation of these compounds and is suitable for large-scale clinical and epidemiological trials as well as smaller sample numbers. A LC-MS/MS method was developed using electrospray ionisation followed by a simple and rapid sample preparation involving enzymatic hydrolysis but without the need to use SPE- or LLE-type extraction methods.

## 2. Materials and Methods

### 2.1. Materials

Taxifolin, daidzein, genistein, and equol were bought from Extrasynthese (France), and acetic acid was obtained from Fluka (UK). Methanol HPLC grade was bought from Fisher Scientific (UK), and sodium dihydrogen orthophosphate was obtained from BDH (Analar). All other chemicals and enzymes for hydrolysis reaction were bought from Sigma (UK).

### 2.2. Human Study

The human intervention study rationale, design, and other details were reported previously [30] and was approved by a National Research Ethics Committee (www.Clinicaltrials.gov: NCT00677599). This trial sought to assess the effects of dietary intervention with soy isoflavones and cocoa flavanols on biomarkers of cardiometabolic risk in postmenopausal women with type-2 diabetes, which is a group of the population at particularly high risk of cardiovascular disease. A urine sample was provided by participants at baseline and after 6 and 12 months of consuming 92 mg of isoflavones (and 90 mg (–epicatechin) contained in two small chocolate bars (total = 27 g chocolate) daily for 12 months. The composition of isoflavones in the chocolate bars was as follows (*μ*mol (g chocolate)^−1^): genistin 1.57; genistein 0.07; daidzin 4.80; daidzein 0.57. Dietary restrictions limited the consumption of specific flavonoid-rich foods prior to baseline sample collection.

### 2.3. Initial Method for Preparation of Urine Samples (Method 1)

The initial urine sample preparation was described in the previous study [31] in Section 2.5. Briefly urine (200 *μ*l) was mixed with phosphate buffer (200 *μ*l; pH 5.0) and taxifolin (10 *μ*l; 10 *μ*g/ml) as an internal standard, before enzyme hydrolysis. Afterwards, a mixture of *β*-glucuronidase (20 *μ*l; 10,000 U/ml (∼200 U)) and sulfatase (20 *μ*l; 1000 U/ml (∼20 U)) (*H. pomatia* types H-5 and H-1, respectively) was added and incubated for 2 h at 37°C for enzymatic hydrolysis. After incubation, DMF (450 *μ*l) and formic acid (40 *μ*l) were added. Samples were allowed to equilibrate for 10 min with a vortex step (30 s) after 5 min and were then centrifuged (13,000 rpm × 15 min, −3°C) prior to HPLC analysis.

### 2.4. Improved Method 1 of Urine Preparation

To improve method 1, various reaction conditions were tested as follows: (1) incubation times from 30 min to 2 h; (2) amounts of *H. pomatia* sulfatase enzyme from 20 up to 400 U and *β*-glucuronidase from 200 to 4000 units per sample; (3) increased pH of buffer up to 6.8 (nearer to the optimum pH for the enzymes); (4) various sulfatase activities were tested–*H. pomatia* types H-1 and H-2, *P. vulgata* (keyhole limpet) types IV and V, *A. aerogenes* type VI, and abalone entrails Type VIII from a variety of different sources.

### 2.5. Final Sample Preparation Method (Method 2)

Urine samples (200 *μ*l) were transferred into 2 ml crew top tubes and mixed with 200 *μ*l phosphate buffer (pH 6.8), sulfatase (80 *μ*l; 1000 U/ml), 80 *μ*l *β*-glucuronidase (80 *μ*l; 10000 U/ml), and internal standard taxifolin (10 *μ*l; 10 *μ*g/ml). After mixing for 1 min, the samples were incubated at 37°C for 2 hours. After incubation, 570 *μ*l DMF and 40 *μ*l formic acid were added and mixed. Samples were equilibrated for 10 min and subsequently mixed every 5 min. After centrifugation for 15 min at 3°C at 13000 rpm, the supernatant was injected (10 *μ*l) onto the HPLC column.

### 2.6. LC-MS Conditions

An Applied Biosystems (AB) Sciex 4000-Qtrap LC-MS/MS mass spectrometer (Applied Biosystems, PE Sciex, Concord, Ontario, Canada) and an Agilent 1200 HPLC system including a pump (binary), degasser, autosampler, autosampler cooler, DAD detector, and column oven (Agilent Technologies, Waldbronn, Germany) were used for the LC-MS analysis. A Phenomenex C18, 3 *μ*m (150 × 3 mm), column was used with a gradient mobile phase with a flow rate of 0.25 ml/ml. Mobile phase A was 13 mM ammonium acetate buffer adjusted to pH 4 with 0.1% acetic acid and B was methanol +0.1% acetic acid. Mobile phase B was increased over 5 min from 0 to 60%, then over 10 min up to 70%; after a 4 min column wash with 100% B, the column was reequilibrated to 0% B for 4 min. Before using, mobile phase A was filtered through 0.65 pore size cellulose nitrate membrane filters (Millipore) using a Millipore vacuum filtration apparatus. Column temperature was 40°C. HPLC flow of 0.25 ml/ml was directly connected to an electrospray ionisation source. Multiple reaction monitoring (MRM) mode and negative polarity were used for the analyses. All authentic reference standards were made in 50% methanol and ammonium acetate buffer and infused with a Harvard Apparatus Syringe Pump at a flow rate of 10 *μ*l/min to optimise the (MRM) parameters: precursor ions (Q1), product ions (Q3), declustering potential (DP), collision energy (CE), and the collision cell exit potential (CXP). The dwell time was set 150 ms for each analyte, and the collision energies (CE) were in the range of −20 to −50 (volts), DP values between −75 to −95 (volts), and CXP values were −7 to −13 (volts). All MRM parameters are shown in Table 1. The source was operated with the nebuliser gas (ion source gas 1 and gas 2) set to 45 psi. The ionisation voltage was set to −4500 V, the entrance potential for all targeted analytes was set at −10 V, curtain gas was to set 25 psi, nitrogen was used as the collision gas and was set to medium, and temperature was 550°C. Analyst software version 1.5 was used for further data processing.

### 2.7. Method Validation

First, baseline urine samples from 15 subjects were extracted and analysed to check for the presence of daidzein, genistein, or equol that could be present because of participants consuming soy via bakery products or other sources of soy flour that were not restricted in the run-in to the intervention period. Urine samples with no detectable or very low levels of isoflavones/equol were selected and used to determine assay precision.

#### 2.7.1. Linearity

Daidzein, genistein, and equol authentic standards were spiked over the range of 0.05–20.0 *μ*g/ml (daidzein and equol) and 0.05–5 *μ*g/ml (genistein) in human urine to construct calibration curves. Linear regression analysis was performed by plotting the concentration verses analyte/internal standard peak area ratio.

#### 2.7.2. Sensitivity

Authentic standards diluted in matrix were individually injected to measure LOD and LOQ values. LOD was calculated as the signal-to-noise ratio 3 times higher than the baseline noise. LOQ was calculated at a signal-to-noise ratio 10 times higher than the baseline noise.

#### 2.7.3. Precision and Accuracy

Intraday precision was estimated by replicate (*n* = 6) analysis of a single pooled human urine sample, following enzymatic hydrolysis. The relative standard deviation was used to assess precision. The interday precision and accuracy of the method were calculated by analysing the same sample duplicates for 10 days.

The bioanalytical precision and accuracy of this method were also calculated by repeating the analysis using samples containing known amount of daidzein, genistein, equol, and taxifolin (internal standard) prepared in blank human urine. Spiking standards in urine (low 0.05 *μ*g/ml (*n* = 2), medium 0.5 *μ*g/ml (*n* = 2), and high 5 *μ*g/ml (*n* = 2)) for genistein, for daidzein, and for equol (low 0.1 *μ*g/ml (*n* = 2), medium 1.0 *μ*g/ml (*n* = 2), and high 10 *μ*g/ml (*n* = 2)) were analysed over 10 days for interday precision, and 10 replicates of 3 levels (low, medium, and high) spiking standards were analysed for intraday precision.

#### 2.7.4. Carry-Over Effect

Blank methanol was injected after an injection of the highest concentration of standards to assess carry over. An Agilent 1200 series high-performance auto sampler with an injection program was used to minimize carry-over effects.

#### 2.7.5. Matrix Effects and Recovery

The postextraction spike method as indicated by the RSC guideline for LC-MS measurements [32] was used to assess the matrix effect. Known concentration of analytes was spiked into two matrixes: (1) 70% methanol and (2) extracted blank human urine, and the peak areas were compared to assess the matrix effect. For recovery assessment, the same procedure was followed except analytes were spiked before the extraction of blank urine.

#### 2.7.6. Stability Study

The room temperature exposure was studied by analysing six replicates of low-spiked QC, high-spiked QC, and also an authentic volunteer sample for each analyte for 6 h; the auto sampler stability was studied by analysing 3 levels of QC samples at the beginning, middle, and end of each batch of sample run. In addition, the system suitable sample, the internal standard, and an authentic volunteer sample (*n*−2) were analysed in each batch of sample analysis to check autosampler stability for 18 h. The freeze thaw stability was not performed because stock solutions and human urine samples were aliquoted and stored at −80°C. An aliquot was analysed each time. For long time storage at −80°C, daidzein, genistein, equol, and taxifolin in methanol were stable for 30 days. Another study [15] also found daidzein and equol were stable in methanol at −70°C for 30 days. All storage conditions were stable because the accuracy and precision was ≤15%, within the acceptable limits.

## 3. Results and Discussion

The present study was designed to develop and validate a simple and rapid method for the quantification of total daidzein, genistein, and equol in human urine samples.

### 3.1. Optimisation of the LC-MS/MS Determination

Reversed-phase liquid chromatographic and MS/MS parameters were optimised to achieve maximum efficiency and sensitivity and short LC run times. Daidzein, genistein, and equol were first characterised by individual infusion of standard solutions (1 *μ*g/ml in ammonium acetate : methanol (50 : 50)) at a flow rate of 10 *μ*l/min into the mass spectrometer in the MRM mode to obtain Q3 ions and some instrumental parameters: DP, CE, and CXP values. Both electrospray (ESI) and atmospheric pressure chemical ionisation (APCI) and positive and negative ion modes were assessed to in order to optimise daidzein, genistein, and equol generate response. The data showed that ESI-MS in the negative ion mode was optimal. These findings are in agreement with the published methods [8]. Under these experimental conditions, other parameters such as drying gas temperature and flow, nebuliser pressure, and capillary voltage were optimised by flow injection analysis (FIA).

To obtain a good separation and the best peak shape, the chromatographic conditions were studied. First, several HPLC separation conditions using different mobile phases were assessed: (i) methanol as the organic phase and ammonium acetate (13 mM) as the aqueous phase with 0.1% acetic acid as an additive in both solvents; (ii) acetonitrile as the organic mobile phase, water as the aqueous phase, and 0.1% formic acid as an additive in both solvents. Better peak shape and separation were obtained by using an aqueous phase of ammonium acetate (13 mM, pH = 4, adjusted by acetic acid) and an organic mobile phase with methanol plus 0.1% acetic acid on C18 (150 × 3 mm), 3 *μ*m column. All our designated compounds were separated with high selectivity. All steps including cleaning and re-equilibration were completed in a relatively short time (≈20 min).

### 3.2. Characterisation of Daidzein, Genistein, and Equol Metabolites in Human Urine

We used urine samples obtained from subjects 24 hours after they had consumed two small chocolate bars containing 92 mg of isoflavones. Enzymatically hydrolysed and nonhydrolysed urine samples were analysed using LC-MS/MS and MRM to detect all the metabolites described in previous studies [8, 33, 34]. The *m/z* values of the investigated isoflavone metabolites in negative polarity are summarized in Table 1. Ten main metabolites were identified by our chromatographic method in nonhydrolysed human urine samples. The main metabolites and LC-MS/MS detection conditions are shown in Table 1.

### 3.3. Study of the Enzymatic Hydrolysis of Daidzein, Genistein, and Equol Metabolites

**Figure 1:**
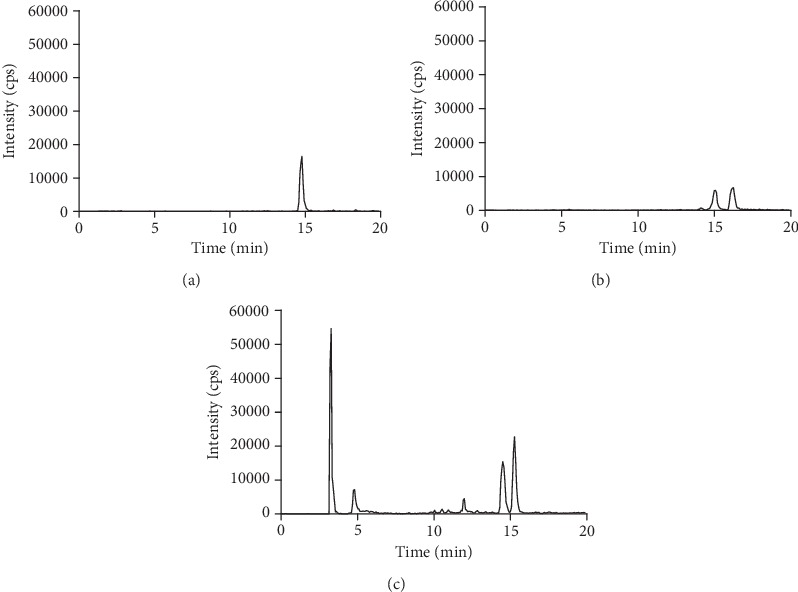
Daidzein, genistein, and equol sulfate metabolites after hydrolysis using a glucuronidase/sulfatase mixture (200/20 U) for 2 h at pH 5 and 37°C. LC-MS/MS extracted ion chromatograms of (a) 333/253 (daidzein sulfate), (b) 349/269 (genistein sulfate), and (c) 321/241 (equol sulfate).

It has been reported that epicatechin sulfates in urine were not hydrolysed after incubation at pH 5 with 200 U of *β*-glucuronidase and 20 U sulfatase for 2 h [31]. Therefore, daidzein, genistein, and equol sulfate metabolite peaks were monitored using LC-MS/MS in enzyme-hydrolysed urine samples. Daidzein, genistein, and equol sulfates were not completely hydrolysed by method 1. The MRM conjugate transitions monitored were *m/z* = −333/−253 (daidzein monosulfate), *m/z* = −349/−269 (genistein monosulfate), and *m/z* = −321/−241 (equol monosulfate) in enzyme-hydrolysed samples (Figure 1). Significant peaks present in the chromatograms of urine samples after enzyme hydrolysis were daidzein sulfate (RT = 14.8 min; *m/z* = −333/−253), two genistein sulfates (RT = 15.04 and 16.22 min; *m/z* −349/−269), and apparently, four equol sulfates (RT = 3.24, 4.78, 14.51, and 15.27 min; *m/z* −321/−241). However, the early peaks (RT = 3.24 and 4.78) with a *m/z* −321/−241 response were, in fact, unlikely to be equol sulfates because their retention times were not consistent with any other isoflavones (14–16 mins) and they were completely insensitive to hydrolysis by sulfatase. Peaks matching daidzein monoglucuronides (*m/z* −429/−253), genistein monoglucuronides (*m/z* −445/−269), and equol monoglucuronides (*m/z* −417/−241) were present in the nonhydrolysed urine samples. These peaks did not appear in the hydrolysed samples. Ion currents for *m/z* transitions *m/z* −253/−91 (daidzein), *m/z* −269/−133 (genistein), and *m/z* −241/−121 (equol) corresponding with the retention times of authentic standards of daidzein (RT = 14.31), genistein (RT = 15.49), and equol (RT = 14.94) were observed in the hydrolysed samples. In nonhydrolysed samples, small ion currents for daidzein and genistein aglycones were present, but equol was not detected. These data suggest that (i) levels of the aglycone forms of daidzein and genistein increased and an equol peak appeared after enzyme hydrolysis of the urine samples, (ii) glucuronide conjugates were efficiently hydrolysed to the corresponding aglycones, and (iii) the hydrolysis of sulfate conjugates was not complete under the conditions used. We estimated the overall degree of sulfate hydrolysis to be around 50%. Taylor et al. have reported complete hydrolysis of isoflavone conjugates in 2 h (37°C, pH 5) [9]. They used >10 times higher enzyme than the amount used in the present study for the same volume of urine (200 *μ*l). In addition, we used type-5 *β*-glucuronidase from *H. pomatia*, whereas Taylor et al. [9] used type HP-2. The different amounts and type of enzyme may have contributed to our distinct observations in the enzymatic hydrolysis reaction.

### 3.4. Tested Alternative: Commercially Available Sulfatases

*H. pomatia* type-1 aryl sulfatase enzyme is commonly used for the hydrolysis reaction. However, we showed that it failed to fully hydrolyse all isoflavone sulfates under the conditions of method 1 (pH 5 for 2 h at 37°C). The other commercially available sulfatases were investigated. A 24 h urine sample containing isoflavones, obtained after consumption of two small chocolates, was hydrolysed with 200 U *β*-glucuronidase and 20 U sulfatase. Each of the commercially available sulfatases: *H. pomatia* types H-1 or H-2, *Patella vulgata* types IV or V, *A. aerogenes* type VI, or abalone entrails type VIII was examined for its ability to hydrolyse isoflavone sulfate conjugates. After analysing the hydrolysed products by LC-MS/MS in each case, all daidzein, genistein, and equol glucuronides had disappeared. This finding indicated that the glucuronides of daidzein, genistein, and equol were completely digested by 200 U *β*-glucuronidase. However, none of the commercially available sulfatases was able to completely hydrolyse sulfate conjugates of isoflavones, as evidenced by the presence of signals at the corresponding retention times for all the sulfate conjugates in posthydrolysed samples (Figure 1).

### 3.5. Improvement of the Hydrolysis of Sulfated Metabolites

**Figure 2:**
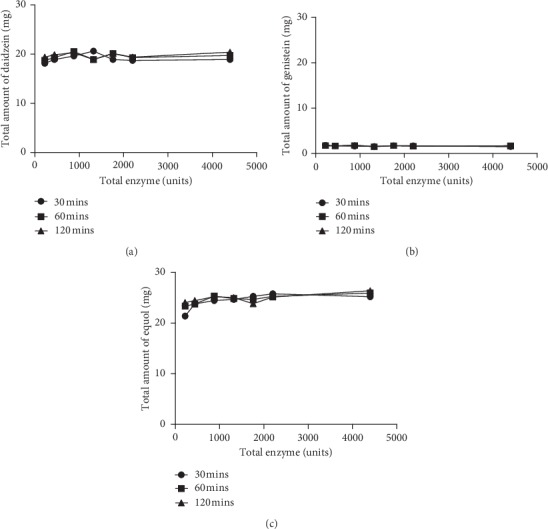
Total daidzein, genistein, and equol amount (mg) plotted against a range of total enzyme units between 220 and 4400. (a) Data 1, daidzein; (b) data 2, genistein; (c) data 3, equol. Note: incubations were carried out over 30, 60, and 120 min at pH 5 and 37°C.

Because daidzein, genistein, and equol sulfate peaks still remained after hydrolysis using method 1 conditions, different conditions were tested. First, daidzein, genistein, and equol conjugates contained in urine samples were treated with a high amount of the *β*-glucuronidase and sulfatase enzymes (*H. pomatia* type H-5 and type H-1, respectively). The total enzyme units were between 220 and 4400 (1 : 10 sulfatase/*β*-glucuronidase ratio), and the samples were incubated at 30, 60, and 120 min at pH 5 and 37°. The daidzein, genistein, and equol aglycone levels after hydrolysis were assessed by LC-MS/MS. Quantification was performed by authentic standards. None of the concentrations of isoflavone aglycones were increased after adding more enzyme or after using longer incubation periods (Figure 2). These data indicated that the *H. pomatia* type H-5 *β*-glucuronidase enzyme can efficiently hydrolyse all daidzein, genistein, and equol glucuronides, but not sulfate conjugates.

Therefore, we investigated the efficiency of hydrolysis at higher pH (6.8). The hydrolysis of isoflavone phase-2 conjugates is usually performed using commercially available enzymes at pH 5 in urine. However, the hydrolysis rate of isoflavone conjugates is higher at pH 6 than pH 5.0 as reported previously [9]. In the present study, all isoflavone conjugates in human urine samples obtained from subjects recruited into the human study described above were completely hydrolysed at pH 6.8 (method 2), despite incomplete hydrolysis at pH 5 (method 1).  Table 2 shows the hydrolysis results between the two methods. Hydrolysis at pH 6.8 was performed as follows: (1) addition of buffer (pH 6.8); (2) addition of internal standard; (3) incubation for 2h with sulfatase (880 U); (4) addition of acid and DMF (to ensure daidzein, genistein, and equol were fully in solution); and (5) centrifugation of the sample. Addition of DMF was supported by a previous report describing catechin extraction [31] and was in keeping with the isoflavone supplier's (Cayman Chemicals) instructions recommending this solvent for dissolving daidzein, genistein, and equol. This extraction method is simple and rapid allowing the processing of a batch of samples, standard, and QC samples in 3-4 h.

### 3.6. Validation of the Proposed Method for Quantification of Total Daidzein, Genistein, and Equol in Human Urine

To confirm identification and quantification of the targeted compounds, a validation method was carried out to establish the performance characteristics of the analytical method. Different parameters such as linearity, sensitivity, precision and accuracy, and limit of detection (LOD) and quantification (LOQ) were determined. The validation data are shown in Table 3. We also compared the daidzein and genistein concentrations obtained with a standard addition calibration curve method and a spiking standard (matrix match) calibration curve method (see Table 4). The results show that our daidzein value was <12% and our genistein value <8%, lower than the spiking standard (matrix match) calibration curve method. The recoveries of all analytes were >90%. The matrix effects were all <10%. Carry-over was shown to be <0.5% for daidzein and <0.3% for genistein and was not detectable for equol.

### 3.7. Application of the Method

The total excretion of daidzein at 6 and 12 months was 75.0 ± 32.7 and 77 ± 5.337 *μ*mol/day, respectively (mean excretion = 52% of ingested dose). The total excretion of genistein at 6 and 12 months was 8.63 ± 4.18 and 9.35 ± 5.93 *μ*mol/day, respectively (mean = 24.9%). Thirty-six percent of the participants receiving the treatment were equol producers (>68.3 *μ*mol/day). The total excretion of equol within equol producers at 6 and 12 months was 59.9 ± 22.1 and 76.6 ± 38.1 *μ*mol/day, respectively (mean = 46.7%).

## 4. Conclusions

In this paper, we report a quantitative LC-MS/MS methodology for the analysis of total daidzein, genistein, and equol in human urine samples that is significantly simpler and quicker than existing and widely used protocols [14]. This is largely because laborious extraction procedures such as SPE, LLE, and sample drying are not required. The method has been shown to possess very good performance characteristics in terms of precision, linear range, sensitivity, and repeatability. It also offers good accuracy, not least due to its ability to completely hydrolyse all tested isoflavone sulfates. The present method is suitable for analysing large numbers of samples, for example, that may be obtained in large cohort epidemiological studies or dietary intervention studies investigating relationships between isoflavone consumption and health outcomes/disease risk. The method reported here is for 200 *μ*L urine samples, but it would be straightforward to scale down and use a UPLC column which would facilitate even shorter run times.

## Figures and Tables

**Table 1:** Summary of the monitored protonated ions and the optimised MS operating parameters of the analytes.

Analytes	(M-H)^−^ (*m/z*)	Precursor ion (*Q*1)	Product ion (*Q*3)	DP (volts)	CE (volts)	CXP (volts)
Taxifolin (IS)	303	303	125	−80	−30	−9
Equol	241	241	121	−75	−20	−7
Genistein	269	269	133	−90	−42	−9
Daidzein	253	253	91	−95	−50	−13
Equol-glucuronide	417	417	241	−75	−20	−7
Equol-diglucuronide	593	593	241	−75	−20	−7
Equol-sulfate	321	321	241	−75	−20	−7
Equol-disulfate	401	401	241	−75	−20	−7
Genistein-glucuronide	445	445	269	−90	−42	−9
Genistein-diglucuronide	621	621	269	−90	−42	−9
Genistein-sulfate	349	349	269	−90	−42	−9
Genistein-disulfate	429	429	269	−90	−42	−9
Daidzein-glucuronide	429	429	253	−95	−50	−13
Daidzein-diglucuronide	605	605	253	−95	−50	−13
Daidzein-sulfate	333	333	253	−95	−50	−13
Daidzein-disulfate	413	413	253	−95	−50	−13
Daidzein-sulfoglucuronide	509	509	253	−95	−50	−13
Genistein-sulfoglucuronide	525	525	269	−90	−42	−9
Equol-sulfoglucuronide	497	497	241	−75	−20	−7

**Table 2:** Comparison of the efficiency of conjugate hydrolysis between method 1 and method 2.

Method name	Daidzein glucuronide (*m/z*: 429/253	Genistein glucuronide (*m/z*: 445/269)	Equol glucuronide (*m/z*: 417/241)	Daidzein monosulfate (*m/z*: 333/253)	Genistein monosulfate (*m/z*: 349/269)	Equol monosulfate (*m/z*: 321/241)
Method 1	−	−	−	+	+	+
Method 2	−	−	−	−	−	−

Method 1 is the original method and does not result in complete hydrolysis of isoflavone and equol sulfates. Method 2 is the new method reported here which efficiently hydrolyses all the isoflavone and equol phase-2 conjugates including the sulfates. + denotes presence of compound after hydrolysis (i.e., not fully hydrolysed) and − denotes fully hydrolysed (i.e., no detectable conjugates observed after hydrolysis).

**Table 3:** Validation data for each analyte in human urine.

Compound	*R* ^2(a)^ (*n* = 10)	Slope^(b)^ (*n* = 10)	Precision, intraday (%)^(c)^	Precision, interday (%)^(d)^	Precision in spiked sample (*n* = 10) (intraday), RSD (%)			Precision in spiked sample (*n* = 10) (interday), RSD (%)			Accuracy (%)			LOD (ng/ml)	LOQ (ng/ml)
					*L*	*M*	*H*	*L*	*M*	*H*	*L*	*M*	*H*		
Daidzein	0.995	2.763	5.9	8.15	9.0	6.37	5.7	19	14.8	11	>100	>90	>90	1	3
Genistein	0.996	0.009	7.2	7.3	4.73	4.1	2.80	17.8	14.1	8.9	>100	>90	>90	1	3
Equol	0.997	3.189			9.24	5.33	3.33	19.1	14.8	8.5	>100	>90	>90	2	6

^(a)^ *R* ^2^ for calibration curve (a measure of goodness of fit of the least squares linear regression); values are mean of 10 calibration curves. ^(b)^Values are mean of 10 calibration curves; units are peak area ratio (*μ*g/ml)^−1^. ^(c)^Precision (determined in an authentic volunteer sample, *n* = 6 replicate analyses); value is relative standard deviation (RSD). ^(d)^Precision (determined in an authentic volunteer sample, *n* = 10 analyses on different days); value is RSD. L: low-concentration quality control sample; M: medium-concentration quality control sample; H: high-concentration quality control sample; LOD: limit of detection; LOQ: limit of quantification.

**Table 4:** Comparison of the quantification of daidzein and genistein using the standard addition and matrix matched calibration curve method.

Compound name	Standard addition method (*μ*g/ml)	Matrix matched calibration curve (*μ*g/ml)
Daidzein	10.5	9.3
Genistein	0.756	0.707

## Data Availability

The workup data used to support the findings of this study are included within the article. The raw data used to support the findings of this study are HPLC chromatograms and peak integrations from diode array and MS detectors connected to the HPLC systems, and these data will be released by the corresponding author upon request (paul.kroon@quadram.ac.uk).
